# Expresser phenotype determines ABO(H) blood group antigen loading on platelets and von Willebrand factor

**DOI:** 10.1038/s41598-020-75462-2

**Published:** 2020-10-27

**Authors:** Diarmaid O’Donghaile, P. Vincent Jenkins, Rachel T. McGrath, Lisa Preston, Stephen P. Field, Soracha E. Ward, Jamie M. O’Sullivan, James S. O’Donnell

**Affiliations:** 1 Irish Blood Transfusion Service, Dublin, Ireland; 2 Department of Haematology, Trinity College Dublin, Dublin, Ireland; 3 Department of Haematology, University Hospital of Wales, Cardiff, Wales UK; 4 National Coagulation Centre, St James’s Hospital, Dublin, Ireland; 5 Cancer Molecular Diagnostics, Trinity Centre for Health Sciences, St James’s Hospital, Dublin, Ireland; 6 Irish Centre for Vascular Biology, School of Pharmacy and Biomolecular Sciences, Royal College of Surgeons in Ireland, Ardilaun House 111 St Stephen’s Green, Dublin 2, Ireland; 7 National Children’s Research Centre, Our Lady’s Children’s Hospital, Dublin, Ireland

**Keywords:** Haematological diseases, Vascular diseases

## Abstract

ABO blood group is associated with cardiovascular disease, with significantly lower risk in blood group O individuals. ABO(H) blood group determinants are expressed on different glycoproteins on platelet surfaces. In addition, ABO(H) structures are also present on VWF glycans. These ABO(H) carbohydrates influence both platelet and VWF function. Previous studies have reported that approximately 5–10% of normal blood donors express abnormally high or low levels of A or B blood group antigens on their platelet surfaces (high expresser phenotype, HXP or low expresser phenotype, LXP respectively). In this study, the biological effects of the ABO Expresser phenotype were investigated. ABO(H) expression on platelets and plasma VWF was studied in a series of 541 healthy blood donors. Overall, 5.6% of our study cohort were classified as HXP, whilst 4.4% satisfied criteria for LXP. We demonstrate that genotype at the *ABO* blood group locus plays a critical role in modulating the platelet HXP phenotype. In particular, A^1^A^1^ genotype is a major determinant of ABO high-expresser trait. Our data further show that ABH loading on VWF is also affected by ABO expresser phenotype. Consequently, A antigen expression on VWF was significantly elevated in HXP individuals and moderately reduced in LXP subjects (*P* < 0.05). Collectively, these findings suggest that ABO expresser phenotype influences primary hemostasis though several different pathways. Further studies will be required to define whether inter-individual variations in ABO(H) expression on platelets and/or VWF (particularly HXP and LXP) impact upon risk for cardiovascular disease.

## Introduction

The ABO blood group system was first recognized by Landsteiner in 1900. Subsequent studies have shown that the *ABO* gene locus is located on chromosome 9 and elucidated the genetic basis underlying different ABO groups^1^. In blood group A, B or AB individuals, alleles at the *ABO* locus encode glycosyltransferase enzymes which catalyze addition of either N-acetylgalactosamine (blood group A) or galactose (blood group B) as capping sugar moieties onto preformed glycan structures^1,2^. In blood group O individuals, the *ABO* locus does not encode a functional transferase enzyme. Consequently, these subjects express unmodified H antigen precursor structures. The presence of ABO(H) blood group antigens on the surface of red blood cells, and their clinical relevance in blood transfusion, is well recognized^3^. Importantly however, ABO(H) antigens are also expressed on other human cells types including endothelial cells (EC) and epithelium^4^. In addition, ABO(H) determinants are expressed on platelets, where they have been identified on a variety of different glycoproteins known to play important roles in hemostasis (including GPIb, GpV, GpIIb/IIIa and PECAM-1)^5^. Recent studies have demonstrated that these ABO(H) glycans on the platelet membrane surface significantly influence normal platelet function^5–7^.

In addition to their cellular expression, covalently-linked ABO(H) structures have also been described on a number of plasma glycoproteins, including von Willebrand factor (VWF), factor VIII (FVIII) and α_2_-macroglobulin^8^. ABO(H) expression on VWF has been characterized in detail^9^. Mass spectrometry (MS) studies have confirmed that ABO(H) determinants are present on both the N- and O-linked glycans of human plasma-derived VWF^10–12^. Interestingly, although H antigen is expressed on platelet-derived VWF, there is no A or B antigen expression^13,14^. ABO(H) expression on VWF is important because it has been shown to regulate multiple aspects of VWF biology^15,16^. First, ABO affects plasma levels of the VWF-FVIII complex, with significantly lower levels in blood group O compared to non-O subjects^17^. This difference is likely attributable to enhanced VWF clearance in group O individuals^18,19^, ABO expression on VWF modulates susceptibility to proteolysis by ADAMTS13, with significantly enhanced cleavage in blood group O subjects^20,21^. Third, accumulating recent evidence suggests that ABO antigens on VWF also influence its functional ability to interact with platelet GPIb^6,22^.

Given the biological importance of ABO(H) expression in regulating platelet and VWF function, it is interesting that marked inter-individual variability in quantitative ABH antigen expression has been reported in healthy individuals^23–25^. Furthermore, several studies have described a so called ABO ‘High Expresser Phenotype’ (HXP), wherein some normal donors express unexpectedly high levels of A or B blood group determinants on their platelets^23,24,26^. Although first described in a cohort of Japanese blood donors^26^, HXP has subsequently been identified in other different ethnic groups with a reported population prevalence of approximately 5%^23,24,27^. This high expresser trait has been observed in subjects with each of the non-O blood groups (A, B and AB respectively), and has been shown to constitute a stable characteristic over time in affected subjects^23,24^. Despite the prevalence and potential translational importance of ABO HXP, the molecular mechanism(s) responsible for mediating increased platelet A or B antigen expression in these individuals remains poorly defined. However familial clustering of HXP has been reported, suggesting that inherited factors contribute to its etiology^23^.

In this study, we investigated the relationship between *ABO* genotype, platelet expresser phenotype, and ABO(H) loading on plasma VWF. Our findings demonstrate that expresser phenotype effect is not confined to ABH expression on platelets, but rather also regulates quantitative ABH loading on plasma VWF. Given the importance of ABO in modulating the biological functions of VWF, these findings are not only of scientific interest but also of direct clinical relevance.

## Materials and methods

### Patient enrolment and ABO typing

Blood samples were collected from healthy adult donors attending the Irish Blood Transfusion Service (IBTS). All donors gave informed consent. The St James’ Hospital Research Ethics Committee has approved the study. In total, samples were collected from 231 group A and 310 group O donors over a one year period. All methods were carried out in accordance with relevant guidelines and regulations. Group A donor sub-grouping was determined by standard red cell serology (i.e. donor red cells that failed to agglutinate when incubated with anti-A_1_ were considered A_2_)^24^. For all blood group A subjects, *ABO* genotype was also determined. In brief, genomic DNA was extracted from whole blood using Gentra-Qiagen Autopure (Alameda, CA) as per the manufacturer’s instructions. PCR was then performed to amplify exons 6 and 7 of the *ABO* gene using oligonucleotide primers as previously described^28^. Primers ABO-1 and ABO-2 in conjunction with the restriction enzyme *KpnI* were used to differentiate O^1^ alleles from A^1^ and A^2^ alleles. Primers ABO-3 and ABO-4 in conjunction with the restriction enzyme *PvuII* were used to differentiate A^2^ alleles from A^1^ and O_1_ alleles.

### Platelet A and H antigen expression

Platelet–rich plasma was prepared by centrifugation of whole blood. Platelet count was then measured using a Cell-Dyn 3200 analyser (Abbott laboratories, IS). To assess A antigen expression levels, 10^6^ platelets were incubated with Phycoerythrin (PE)-labeled anti-human CD41a (BD Biosciences Pharmingen; San Diego, CA) and Fluorescein (FITC)-labelled Helix pomatia (HPA, Anti-A) (Sigma-Aldrich; St. Louis, MO) for 30 min as previously described^28^. Samples were then analyzed using a BD FacsCanto II (BD Biosciences, San Jose, CA). Platelets staining dual-positive for both CD41a and anti-A were quantified, together with mean fluorescence intensity for CD41a and HPA expression. Data were quantified using FACSDiva software (BD Biosciences, San Jose, CA). In keeping with previous studies, HXP was defined as greater than 75% platelet A antigen positivity, and LXP defined as less than 15% platelet positivity^23,24^. Similarly, FITC-labeled *Ulex europaeus* (UEA, anti-H) (Sigma-Aldrich; St. Louis, MO) was used to investigate H antigen expression on group O platelets. Finally, platelet α2-6 linked sialic acid expression was analyzed using FITC-labeled *Sambucus nigra agglutinin* (SNA) (Vector Laboratories Inc: Burlingame, CA). For each glycan analysis, a parallel sample was analyzed using isotype control (FITC-labelled mouse IgG1κ isotype; BD Biosciences Pharmingen, San Jose, CA).

### Plasma VWF antigen levels

Plasma VWF antigen (VWF:Ag) levels were measured by sandwich enzyme-linked immunosorbent assay (ELISA) as previously described^29^. Briefly, 96-well ELISA plates (DAKO) were coated with rabbit polyclonal anti-human VWF antibodies (A082; Dako, Denmark) diluted in 0.05 M carbonate buffer (pH 9.6). After washing, the plates were incubated with the test samples or reference plasma. Following further washing, the plates were incubated with rabbit polyclonal anti-human VWF peroxidase conjugate, (P266; Dako, Denmark) for 1 h. VWF:Ag concentration was determined by measuring optical density at 492 nm. Dilutions of Reference plasma were used to construct standard curves for calibration. All ELISA samples were tested in duplicate. The intra-assay and inter-assay coefficients of variation were both less than 5% and the lower limit of VWF:Ag detection was 0.03 IU/mL.

### A antigen expression on plasma VWF

Group A (GalNAc α1 → 3 [Fuc α1 → 2] Galβ 1 → 4 GlcNAc β1 →) antigenic determinants on plasma were measured using a modified sandwich ELISA^28,30^. In brief, ELISA plates were coated with rabbit anti-human VWF (Dako), washed and blocked using TBS containing 1% BSA. After further washings, plasma samples were added and incubated for 2 h at room temperature. Each plasma was tested in duplicate at three dilutions. The plates were washed and then incubated with murine anti–A monoclonal antibody (Ortho Diagnostics) for 1 h. After a further three washes, the plates were incubated with goat anti-mouse IgM peroxidase conjugate (Sigma) for 1 h. After another TBS/Tween wash, peroxidase substrate solution was added. The reaction was stopped with 1 M H_2_SO_4_ and the optical density measured at wavelength 492 nm. Pooled group A plasma was assayed to produce a standard curve for each ELISA. Using the standard curve, a value for A antigen on VWF expression was determined for each plasma sample. Plasma VWF:Ag concentration strongly influenced the amount of A antigen detected in each ELISA well. To determine the amount of A antigen expressed per unit VWF, the amount of A antigen detected was divided by the amount of VWF:Ag present in the ELISA well^28^. The pooled normal A plasma was assigned a value of 1U/ml for the amount of A antigen expressed per unit vWF.

### Statistical analysis

Statistical analysis was performed using GraphPad Prism Version 5.0 (Graphpad Software, San Diego, CA) and statistical significance was assigned at a value of *P* < 0.05. Mann–Whitney U test was used to test differences in mean values.

## Results

### High and low expresser phenotypes in normal Irish blood donors

**Figure 1:**
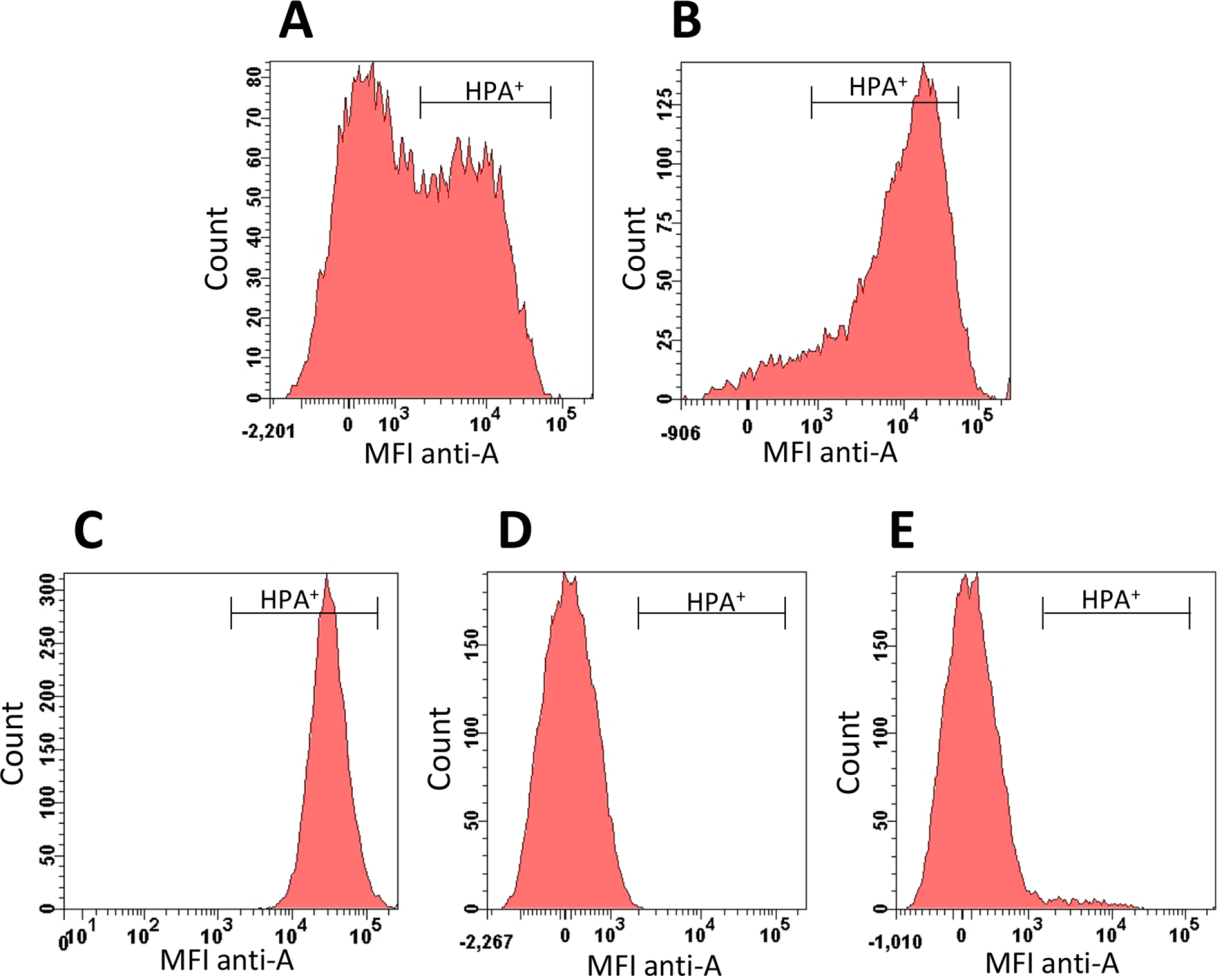
Patterns of A antigen expression on platelets in group A normal donors. Platelet rich plasma was prepared from each group A normal donors. Platelet A antigen expression was then analyzed by flow cytometry using the lectin *Helix pomatia* (HPA). (**A**) The majority of Group A_1_ donors demonstrated a wide distribution including both negative and positive platelet populations. A representative histogram is shown. (**B**,**C**) 10 individuals (5.5%) were classified as group A High Expresser (≥ 75% platelets expressing A antigen). These HXP were further sub-classified on the basis of their platelet histogram distribution appearances. (**B**) 8 individuals were designated as type I HXP and (**C**) 2 donors were designated type II HXP. (**D**) Minimal platelet A antigen expression was seen in blood group A_2_ individuals. A representative histogram is presented. (**E**) Finally, 8 A1 donors demonstrated significantly reduced A antigen expression (≤ 15% platelets expressing A antigen) and were classified as Low Expresser Phenotype (LXP). MFI = mean fluorescence intensity. (**F**) For each of the 231 heathy blood group A subjects recruited, red cell phenotyping was performed to define A_1_ and A_2_ subjects. Thresholds used to define HXP and LXP individuals are highlighted.

231 blood group A subjects were recruited from healthy donors. 182 (79%) of the donors were male and 49 (21%) were female. Red cell analysis confirmed that 180 (78%) donors were phenotypically A_1_, whilst the remaining 51 (22%) subjects were A_2_. For each donor enrolled, blood group A antigen expression on platelets was assessed by flow cytometry. In keeping with previous studies, significant heterogeneity in A antigen expression on platelets was observed even for a given individual subject (Fig. 1A). In particular, a bimodal pattern of A antigen expression on group A platelets was observed in the majority of individuals studied. Previous studies have applied a diagnostic threshold of ≥ 75% platelets expressing A antigen in order to identify individuals with High Expressor Phenotype^23,24^. Using this criterion, 10 (5.6%) of our study cohort were classified as HXP. In keeping with previous findings*,* we observed two distinct subgroups within our HXP cohort. In 8 HXP subjects, the platelet histogram was similar in shape to that of normal A_1_ platelets, but shifted to the right toward higher values (type I HXP) (Fig. 1B). In contrast, 2 HXP subjects had platelet histograms consisting of a single sharp peak (type II HXP) (Fig. 1C). In keeping with previous studies, repeat sampling confirmed that HXP was a consistent finding in affected individuals over time^23,24^. Minimal platelet A antigen expression was seen in blood group A_2_ individuals (Fig. 1D). Finally, 8 A_1_ donors demonstrated significantly reduced A antigen expression (≤ 15% platelets expressing A antigen) and were classified as Low Expresser Phenotype (LXP) (Fig. 1E). Collectively, these data demonstrate that significant intra- and inter-individual heterogeneity in platelet A antigen expression is present amongst healthy subjects. Moreover, a significant proportion of normal donors demonstrate abnormally ABO High or Low Expresser Phenotypes.

### ABO genotype and expresser phenotype

**Figure 2:**
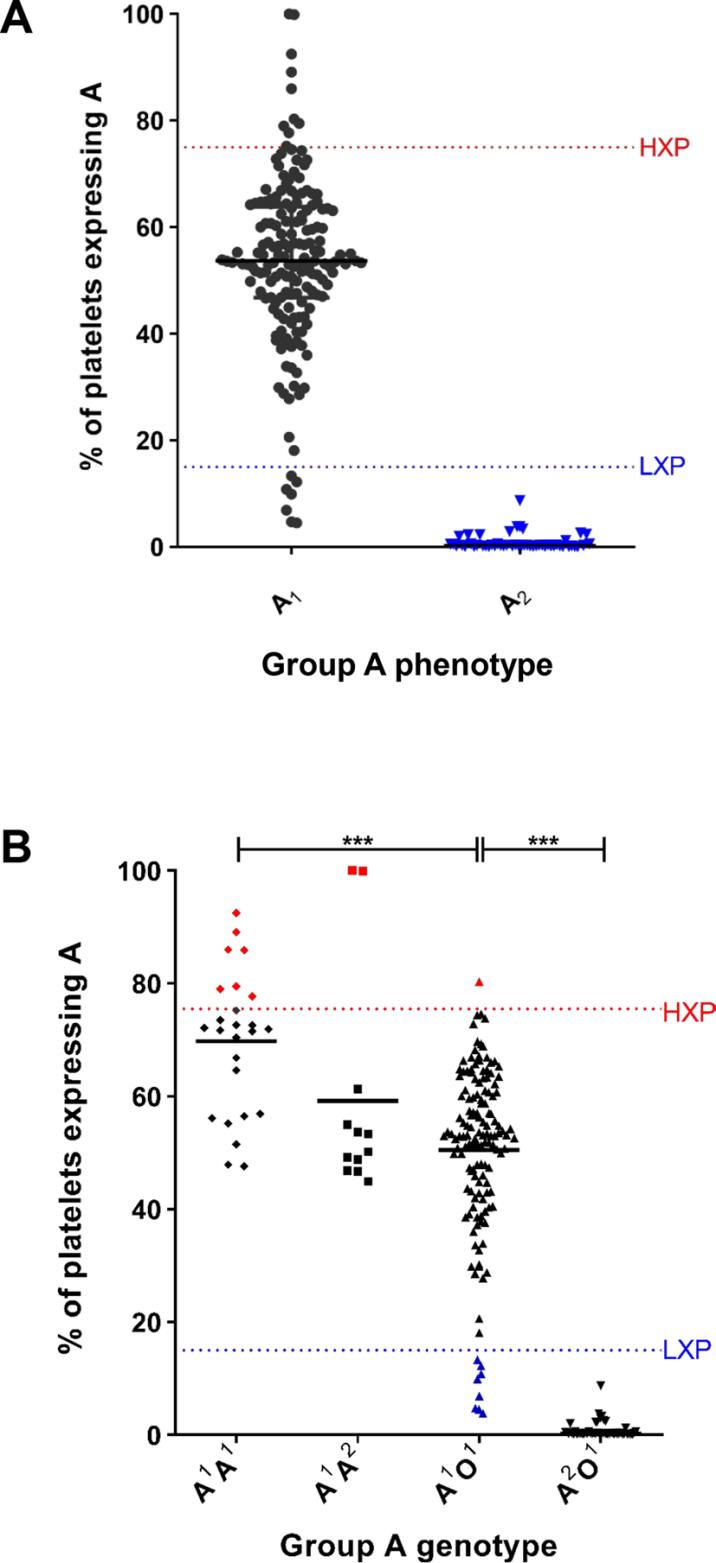
The relationship between *ABO* genotypes, ABO expresser phenotypes and platelet A antigen expression. To investigate whether genotype at the *ABO* blood group locus influences expresser phenotype, PCR–RFLP analysis was performed for all 231 group A donors. Platelet A antigen expression was significantly elevated on homozygous A^1^A^1^ donors (mean 71.9% HPA +) (black diamond) compared to A^1^A^2^ (mean 51.8% HPA +) (black square) or A^1^O^1^ (mean 53.1% HPA +) (black triangle) individuals (*P* < 0.01 and *P* < 0.001, Mann–Whitney). In keeping with previous reports, minimal platelet A antigen expression was observed in group A_2_ subjects. The ABO genotypes of the 10 HXP donors in our cohort are highlighted in red (7A^1^A^1^; 2A^1^A^2^ and 1 A^1^O^1^ respectively). In contrast, all LXP subjects (highlighted in blue) had genotype A^1^O^1^ (***P < 0.001).

In blood group A_2_ individuals, a deletion in the *ABO* gene results in a frame-shift, which significantly reduces the activity of the A transferase enzyme compared to A_1_ subjects^31^. Consistent with this concept, platelet A antigen expression was significantly increased in group A_1_ compared to A_2_ healthy donors and all of the subjects classified as HXP were A_1_ (Fig. 2A). Interestingly however, the amount of A antigen expressed on platelets varied widely between the different individuals (Fig. 2A). Previous studies have demonstrated that amongst A_1_ donors, *ABO* genotype exerts a dosage effect, such that A transferase levels are significantly higher in A^1^A^1^ compared to A^1^O^1^ individuals^28^. Consequently, we further investigated the relationship between *ABO* genotype and expresser phenotype. Using PCR–RFLP analysis, *ABO* gentoype was defined for each of our 231 group A donors. Overall 25 individuals were homozygous A^1^A^1^, 12 subjects were A^1^A^2^ , and 143 donors were A^1^O^1^. Consistent with the red cell phenotyping data, ABO genotyping confirmed that 50 donors were A^2^O^1^ and 1 subject was A^2^A^2^. In keeping with the concept that there is a dosage effect of the *ABO* locus on enzymatic activity, platelet A antigen expression was significantly higher in homozygous A^1^A^1^ compared to heterozygous A^1^O^1^ individuals (*P* < 0.001; Fig. 2B). Furthermore, 7 of the 10 individuals classified as HXP were also found to have genotype A^1^A^1^. Surprisingly, given the limited A transferase activity associated with the A^2^ allele, two of the other HXP donors genotyped as A^1^A^2^ (Fig. 2B). The prevalence of HXP amongst A^1^A^1^ subjects was significantly increased compared to the total A donor population (28% versus 4.3%; *P* < 0.05). In contrast, the prevalence of HXP was markedly reduced in heterozygous A^1^O^1^ donors (0.7%). All the LXP subjects identified in our cohort had an A^1^O^1^ genotype. Together, these data clearly demonstrate a major role for *ABO* gentoype in modulating quantitative A antigen expression on platelets in normal subjects and further suggest that genotype is an important factor in HXP etiology.

### ABO blood group and platelet sialylation

**Figure 3:**
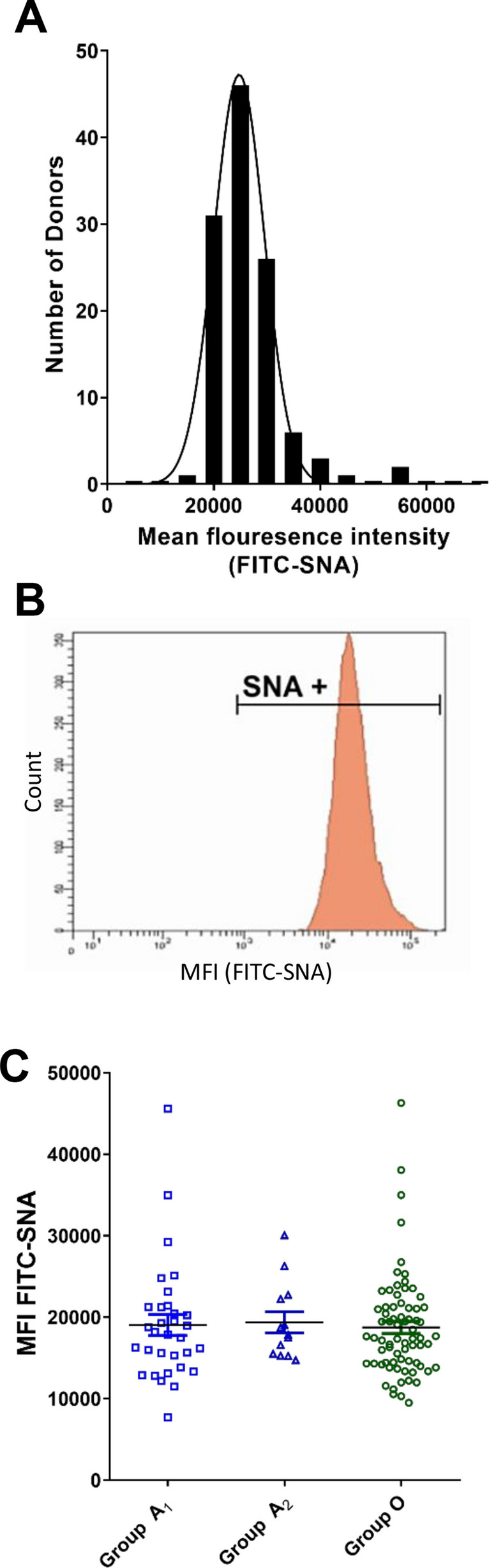
ABO blood group and α2-6 linked sialic acid expression on platelets. Platelet rich plasma was prepared from normal group A and group O donors. Platelet α2-6 linked sialic acid expression was then analyzed by flow cytometry using the lectin *Sambucus nigra agglutinin* (SNA). (**A**) Significant inter-individual variation in SNA mean fluorescence intensity (MFI) was observed between normal donors. (**B**) For individual subjects, platelets were typically all positive for SNA. A representative histogram is presented. (**C**) ABO phenotype had no significant effect on platelet α2-6 linked sialylation.

Recent studies have shown that ABO(H) antigen expression on human erythrocyte surfaces influences α2-6 linked sialic acid presentation by stabilizing sialylated glycan clusters^32^. Consequently, *Sambucus nigra agglutinin* (SNA) lectin binding to erythrocytes was shown to differ significantly between ABO blood groups (A > O > B)^32^. Given these data, we investigated whether there was a relationship between ABO blood group and sialic acid expression on platelets. Similar to A antigen expression, significant inter-individual variability in platelet sialylation was observed (Fig. 3A). However, unlike the bimodal pattern observed for platelet A antigen expression, SNA platelet-binding demonstrated a single positive unimodal distribution (Fig. 3B). The difference in α2-6 linked sialylation compared to A antigen expression on platelets is interesting, since both are characterized by single terminal sugar moieties on the end of complex glycan chains. Critically however, unlike in erythrocytes, no significant relationship between ABO group and α2-6 linked sialylation on platelets was seen (Fig. 3C). Furthermore, we observed no correlation between neither quantitative A or H antigen expression and platelet SNA binding (data not shown).

### Plasma VWF in high and low expressor phenotypes

**Figure 4:**
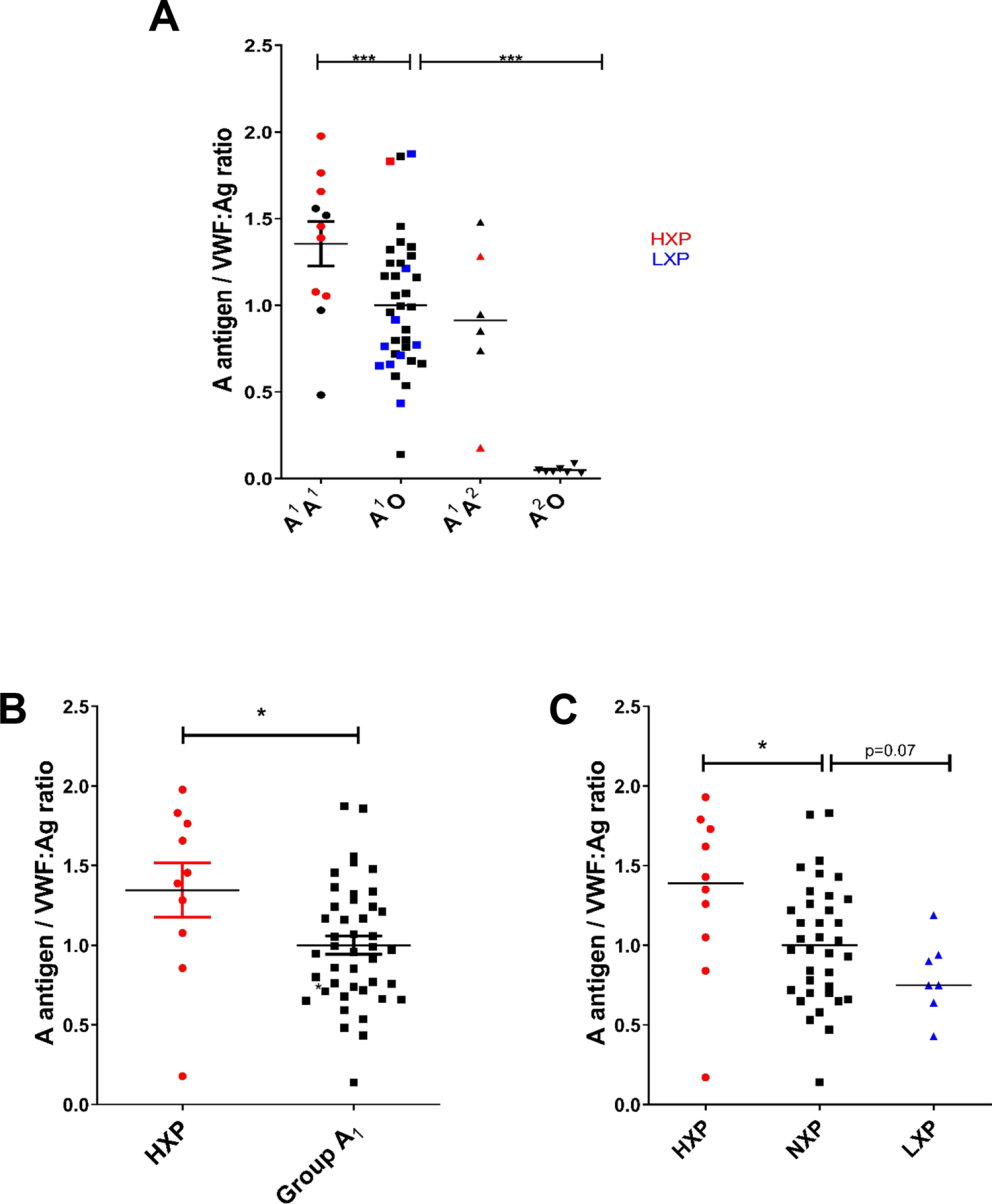
The relationship between *ABO* genotypes, ABO expresser phenotypes and the amount of A antigen loading on plasma VWF. To determine whether expresser phenotype influences ABO loading on plasma VWF, the amount A expressed per unit VWF (AVWF) was quantified in each blood group A donor using a modified sandwich ELISA. All experiments were performed in triplicate, and pooled normal A plasma was assayed to produce a standard curve. (**A**) A antigen expression on plasma VWF was significantly elevated in homozygous A^1^A^1^ donors compared to A^1^A^2^ or A^1^O^1^ individuals. In keeping with previous reports, minimal A antigen expression on plasma VWF was observed in group A_2_ subjects. (**B**) A antigen expression on plasma VWF was significantly increased in HXP subjects compared to normal A_1_ controls (*P < 0.05). (**C**) A antigenic loading on VWF was significantly higher in HXP individuals compared to those with a normal- (NXP) or low- (LXP) expresser traits (*P < 0.05; *** *P* < 0.001).

**Figure 5:**
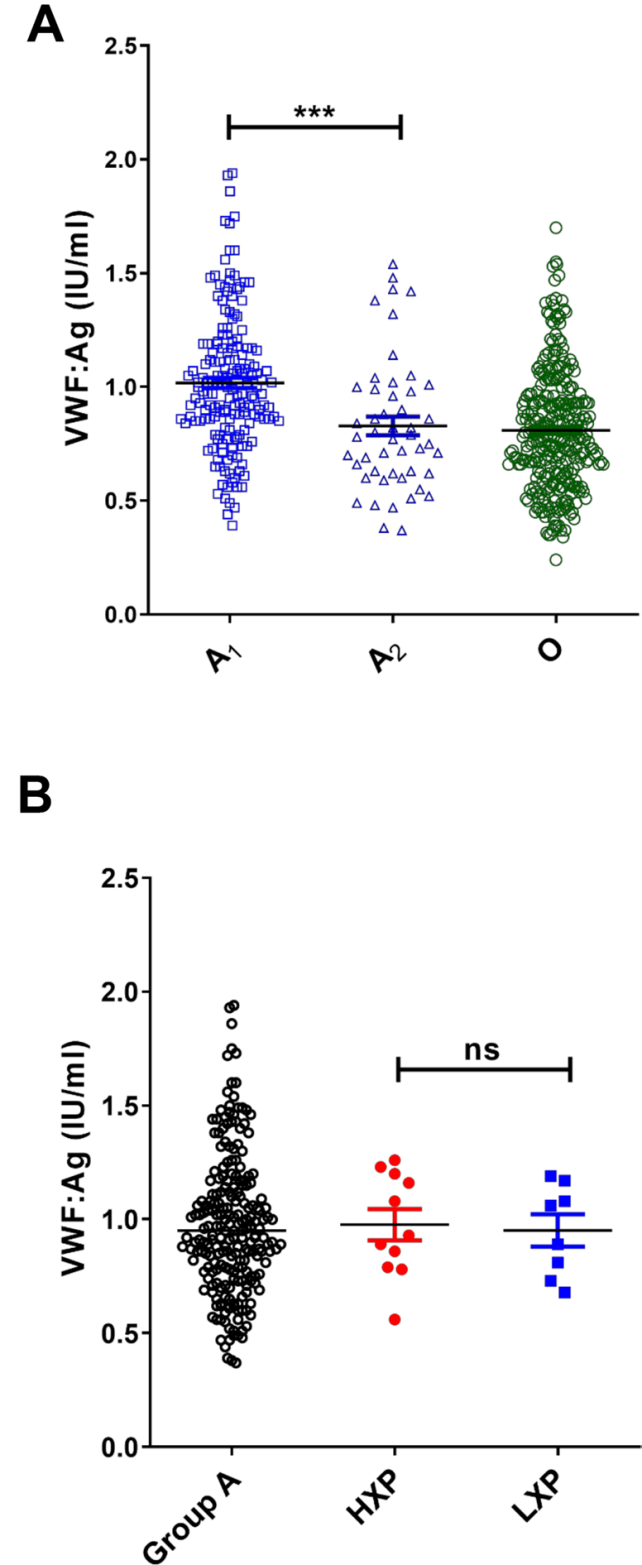
The relationship between ABO phenotypes, ABO expresser phenotypes and plasma VWF:Ag levels. Plasma VWF:Ag levels were measured in each subject using standard ELISA. (**A**) Plasma VWF:Ag levels were significantly higher in A_1_ compared to A_2_ or group O subjects. (**B**) No significant differences in plasma VWF:Ag levels were observed between HXP, LXP and group A donors (***P < 0.001; ns = not significant).

Based upon our *ABO* genotype and H antigen expression data, we hypothesized that increased glycosyltransferase expression may be important in platelet HXP etiology. To further investigate this hypothesis, we next studied whether *ABO* genotype and expresser phenotype had any effect on quantitative loading of A antigen on plasma VWF (AVWF). Using a previously optimized modified sandwich Elisa^28^, AVWF was measured for each individual in our group A cohort. Although marked inter-individual variability in AWF was observed, a significant effect of *ABO* genotype was also seen (Fig. 4A). Consistent with our platelet data, AVWF was significantly increased in homozygous A^1^A^1^ individuals compared to heterozygous A^1^O^1^ subjects and minimal A loading on plasma VWF was observed in A_2_ subjects (Fig. 4A). In addition, a significant relationship between platelet ABO expresser phenotype and quantitative AVWF expression was also observed, with AVWF levels significantly increased in platelet HXP subjects compared to group A individuals (*P* < 0.05; Fig. 4B). Finally, a dosage effect of platelet expresser phenotype on AVWF was seen, with significantly increased in platelet HXP subjects compared to those classified with a normal expresser phenotype (*P* < 0.05; Fig. 4C). Conversely, AVWF was markedly reduced in in platelet LXP compared to HXP individuals (*P* = 0.02; Fig. 4C). Consistent with previous studies, plasma VWF:Ag levels were significantly higher in blood group A_1_ subjects (genotypes A^1^A^1^ or A^1^O^1^) compared to either A_2_ or group O respectively (Fig. 5A). Although A antigen loading on VWF was significantly higher in HXP individuals, no effect on plasma VWF:Ag levels was observed (Fig. 5B).

## Discussion

ABO blood group has been associated with a number of different types of sepsis including *Plasmodium falciparum, Helicobacter pylori, Salmonella typhi* and Covid-19)^33–36^. In addition, accumulating data have demonstrated that ABO blood group also influences risk for cardiovascular disease^37^. Although the mechanisms through which ABO impacts vascular risk remain poorly understood, recent evidence suggests that ABO(H) expression on platelets influences platelet function^5,6,25^. Our data highlight that there is significant inter-individual heterogeneity in platelet A antigen expression, even amongst healthy group A_1_ donors. Furthermore, we also demonstrate that the amount of A antigen carried on circulating platelets varies widely even in a given individual subject. In addition to this inter- and intra-individual variation in platelet ABO(H) expression, approximately 5% of normal Irish A_1_ donors exhibited significantly enhanced A antigen expression on platelets and thus satisfied proposed criteria for ABO high-expresser trait. Conversely, a further 5% of A_1_ subjects demonstrated significantly attenuated A antigen expression on circulating platelets and thus could be classified as ABO low-expresser trait. Despite the fact that our population were almost all Caucasian, it is interesting that these frequencies are remarkably similar to those reported in similar studies performed in other ethnic groups^23,26,27^.

With respect to the relationship between ABO and cardiovascular risk, it is important to emphasize that ABO(H) blood group determinants are expressed on a variety of different glycoproteins and glycolipids on the platelet surface^5^. It remains unclear whether the HXP/LXP effects apply equally to ABO(H) loading on each of these different platelet glycoproteins. Importantly however, previous studies reported that HXP specifically influenced quantitative ABO(H) expression on the platelet GpIb^23^. This observation is interesting because ABO directly influences GpIb functional activity^6^. Using video microscopy, Dunne et al. demonstrated that the ability of group O platelets to interact with immobilized VWF was significantly reduced compared to non-O platelets^6^. Consequently, under arterial shear conditions, type O platelets travelled further and moved at faster translocation velocities before they were able to form stable interactions with immobilized VWF. Subsequent analyses showed that platelet GpIb in group O individuals was less efficient in modulating VWF interaction^6^. Together, these data are consistent with the increased frequency of blood group O individuals amongst patients diagnosed with mild quantitative von Willebrand disease (VWD)^38,39^.

In addition to influencing platelet function, ABO(H) blood group antigens are also expressed on VWF and FVIII in normal human plasma^35^. Importantly in the context of the association between ABO group and vascular disease, ABH determinants expressed on VWF glycans have been shown to influence VWF functional activity; VWF susceptibility to proteolysis and VWF clearance^35^. Thus, in keeping with their reduced thrombotic risk, plasma levels of the VWF-FVIII complex are significantly lower in blood group O compared to non-O individuals. Our findings demonstrate that similar to platelets, ABH expression on plasma VWF also varies widely between normal subjects. For the first time, we further demonstrate that ABH loading on VWF is also affected by ABO expresser phenotype. Consequently, although the number of subjects was limited, A antigen expression on VWF was significantly elevated in HXP individuals and moderately reduced in LXP subjects. Current evidence suggests that addition of ABO(H) determinants occurs during post-translational modification within EC rather than in the peripheral circulation^15,40,41^. Consequently, our findings suggest that the molecular mechanisms underlying HXP and LXP are likely shared across a number of different cell types. Despite the significant effect of high expresser-trait in regulating ABO expression on plasma VWF, we observed no significant effect on plasma VWF:Ag levels. This may be attributable to the small number of HXP individuals in our cohort. Alternatively, it is possible that the relationship between ABO blood group and plasma VWF clearance rate is not regulated via ABH determinants expressed on VWF glycans.

To investigate mechanisms that may contribute to expresser-traits, we performed *ABO* genotyping for subjects in our cohort. A antigenic loading on both platelets and VWF was significantly increased in homozygous A^1^A^1^ compared to heterozygous A^1^O^1^ individuals. In addition, minimal A antigen expression was present on platelets or VWF in A_2_ subjects. Importantly, 7/10 of the individuals with ABO HXP had an A^1^A^1^ genotype. However, the majority of individuals with an A^1^A^1^ genotype did not have evidence of abnormally elevated A antigen expression on either platelets or VWF, and were not classified as HXP. Moreover, 3 HXP subjects were shown to have genotypes A^1^A^2^ or A^1^O^1^ respectively. Collectively, these data highlight that *ABO* genotype is an important factor in determining HXP. Given the known dosage effect of the *ABO* gene^28^. we postulate that increased glycosyltransferase expression represents at least in part the likely causal mechanism^23^. This hypothesis is supported by a previous study that reported elevated glycosyltransferase enzymes in the serum of HXP subjects. Nevertheless, since not all A^1^A^1^ individuals exhibit elevated platelet A antigen expression, clearly additional and as yet unrecognized mechanisms must also be involved in contributing to ABO HXP pathogenesis.

In the majority of A_1_ donors, flow cytometry studies showed a bimodal pattern of A antigen expression on platelets. Thus, in the same individual at the same time point, some platelets demonstrated strong A antigen expression whilst other platelets had low A antigen. Although these findings are consistent with several previous studies^23,24^, the underlying mechanism remains unknown. To gain further insight into factors that might modulate ABO(H) loading on platelets and VWF, we measured α2-6 linked sialic acid expression on platelets. Similar to ABO(H) determinants, α2-6 linked sialic acid is expressed as a terminal capping sugar moiety at the end of glycan antennae^42^. Although recent studies reported a relationship between ABO blood group and terminal sialylation on human red cells^32^, we found no evidence that α2-6 linked sialylation influences quantitative ABO(H) expression on either platelets or VWF respectively.

In conclusion, our findings highlight the fact that ABO expresser traits significantly influence quantitative ABO(H) expression on both platelets and plasma-derived VWF. This concept is important when considering potential biological pathways that may explain emerging GWAS data identifying *ABO* as a disease-associated locus. Additional studies will be required to define whether HXP and LXP also influence ABH expression in other tissues. In addition, further research will also be necessary to elucidate whether inter-individual variations in ABO(H) expression on platelets and/or VWF (particularly HXP and LXP) impact upon risk for cardiovascular disease (e.g. bleeding, thrombosis, TTP)^8,43^, or indeed susceptibility to specific types of sepsis.
